# Cis or trans: a puzzle of Parkin activation mechanism

**DOI:** 10.1042/EBC20253048

**Published:** 2026-02-02

**Authors:** Mohini Sherawat, Ankit Kumar, Dipti Ranjan Lenka, Atul Kumar

**Affiliations:** 1 Department of Biological Sciences, Indian Institute of Science Education and Research (IISER) Bhopal, Bhopal, 462066, India

**Keywords:** mitophagy, Parkinson’s, Parkin, ubiquitin, PINK1

## Abstract

The *PARK2* gene, which encodes the E3 ubiquitin ligase Parkin, and the *PARK6* gene, encoding phosphatase and tensin homolog (PTEN)-induced kinase 1 (PINK1), are frequently mutated in patients with Parkinson’s disease (PD). Parkin is normally maintained in an autoinhibited conformation, and its activation is triggered by PINK1-mediated phosphorylation of both ubiquitin or NEDD8 and Parkin’s ubiquitin-like (Ubl) domain. This review provides a comprehensive overview of the models proposed over the past decade to explain Parkin’s autoinhibition and activation. We summarize key structural and biophysical studies that have progressively uncovered the molecular basis of Parkin activation, tracing the evolution of these insights. This review concludes by discussing the intriguing and still unresolved question of whether Parkin activation occurs through a cis or trans mechanism and outlines future directions for research aimed at understanding these pathways.

## Introduction

Parkinson’s disease (PD) is a neurodegenerative disorder marked by the progressive loss of dopaminergic neurons in the midbrain. While most cases are sporadic, 15–20% of cases form a familial form of PD [1]. Several genes, such as *SNCA*, *PARK2*, *PINK1*, *PARK7*, and *LRRK2,* are associated with autosomal recessive juvenile Parkinsonism (AR-JP) [1–4]. Mutations in the *PARK2 (PRKN*) and *PARK6 (PINK1*) genes account for most of the familial cases, specifically those presenting as autosomal recessive juvenile Parkinsonism (AR-JP) [4–11]. *PARK2* encodes Parkin, an E3 ubiquitin ligase, while *PARK6* encodes PINK1 [10,11]. Notably, *PARK2* mutations alone are responsible for approximately 50% of AR-JP cases, with over 200 different missense and deletion mutations identified in Parkin [7,11–18].

Parkin functions as an E3 ubiquitin ligase, targeting multiple substrates for ubiquitination, which is crucial for protein degradation and maintaining mitochondrial homeostasis [19–23]. This underscores its essential role in cellular integrity and its relevance to the pathogenesis of neurodegenerative diseases.

E3 ubiquitin ligases are generally classified into three main families. RING (Really Interesting New Gene) family E3 ligases use a zinc finger-containing RING domain to recruit E2 ubiquitin-conjugating enzymes and transfer ubiquitin directly to the substrate [24–29]. HECT type (Homologous to E6AP Carboxyl Terminus) E3 ligases possess N and C-lobes; the N-lobe binds E2, while the C-lobe forms a thioester intermediate with ubiquitin before transferring it to the substrate lysine [30–34]. RBR (RING-between-RING) E3 ligases combine features of both: they bind E2 via a RING domain and use a catalytic cysteine residue to form a thioester intermediate before ubiquitin transfer [35–41].

Parkin belongs to the RBR family, which follows a conserved catalytic mechanism across its members, although the regulatory mechanisms can vary significantly [35,36,38–41]. For instance, Parkin is activated by PINK1-mediated phosphorylation [42–48], whereas HHARI, another RBR ligase, is regulated via neddylation, enabling interaction with cullin proteins [49].

**Figure 1:**
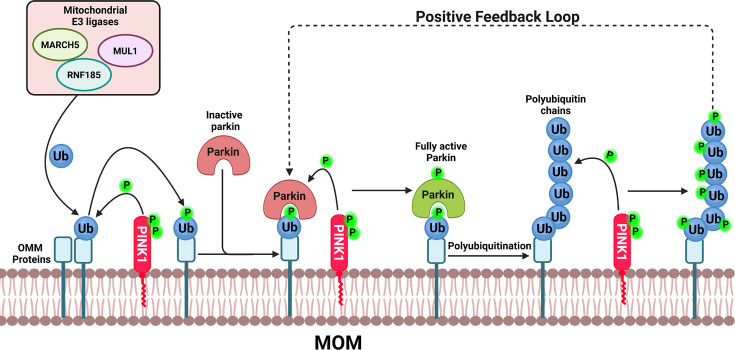
Feedforward mechanism of PINK1-mediated Parkin activation.

PINK1 and Parkin function together to maintain mitochondrial quality in response to cellular stress and mitochondrial damage. Under stress conditions, PINK1 accumulates on the outer mitochondrial membrane, where it phosphorylates serine 65 (S65) on ubiquitin [47,48,50,51] that has been conjugated to outer-membrane proteins by mitochondrial E3 ligases such as MUL1, MARCH5, and RNF185 (Figure 1) [52]. Phosphorylated ubiquitin (pUb) binds with high affinity to cytosolic, autoinhibited Parkin, promoting Parkin’s recruitment to damaged mitochondria [47,48,50,51]. Once localized to the mitochondria, Parkin is phosphorylated at its conserved S65 residue within the ubiquitin-like domain [43,53], thereby fully activating its E3 ligase activity. Activated Parkin then ubiquitinates outer-membrane proteins. PINK1 subsequently phosphorylates these newly attached ubiquitin molecules, creating a positive feedback loop that amplifies Parkin-mediated ubiquitination of mitochondrial proteins (Figure 1) [48,54–56].

In this review, we outline the key discoveries that have elucidated the transition from Parkin’s autoinhibited state to its active form. We trace the evolution of models explaining Parkin activation and conclude with current hypotheses, highlighting open questions, particularly regarding the cis and trans mechanisms of Parkin activation.

## Parkin autoinhibition: the multidomain puzzle

**Figure 2:**
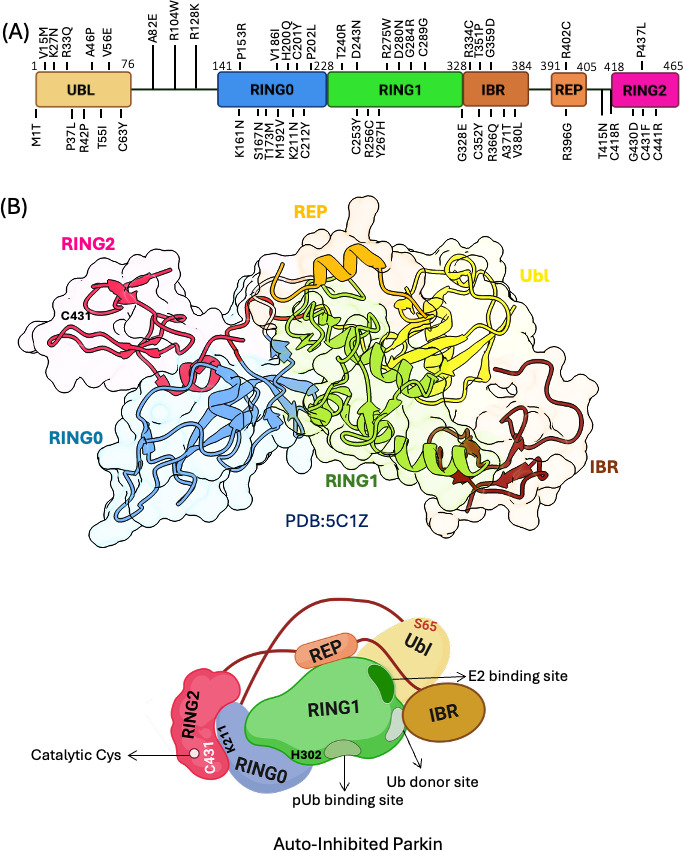
Domain architecture and autoinhibited structure of Parkin. **(A**) Schematic of Parkin’s domain organization, indicating domain boundaries and PD mutations. (**B**) Crystal structure of autoinhibited Parkin (PDB: 5C1Z), showing intramolecular interactions that stabilize the inactive conformation. Lower panel: schematic illustration of Parkin’s autoinhibited state, highlighting key structural features.

Parkin is a member of the RBR (RING-between-RING) family of E3 ubiquitin ligases. Its domain architecture includes an N-terminal ubiquitin-like (Ubl) domain (residues 1–76), followed by a flexible linker (77–140) connecting to the RING0 domain (141–228), which precedes the RBR catalytic module comprising RING1, IBR (in-between RING), and RING2 domains (Figure 2A) [57,58].

The concept of Parkin existing in an autoinhibited state was first proposed by Chaugule and colleagues, who demonstrated that several pathogenic mutations in Parkin disrupt this autoinhibition, resulting in increased enzymatic activity [59]. In the absence of structural data, early biochemical and biophysical studies identified the Ubl domain as a key element in maintaining autoinhibition. Chaugule et al. [59] elegantly showed that titration of the isolated Ubl domain to a ΔUbl Parkin (lacking the Ubl domain and thus constitutively active) could restore autoinhibition. Furthermore, disease-associated mutations within the Ubl domain, such as K27N, R33Q, R42P, and A46P, induced autoubiquitination activity, strongly implicating the Ubl domain is crucial in maintaining Parkin in its inactive state [59,60].

Subsequent structural studies provided detailed insights into the molecular basis of Parkin autoinhibition [57,61,62]. Crystal structures of full-length Parkin revealed that the Ubl domain interacts with RING1, occluding the E2 enzyme-binding site and thereby preventing ubiquitin transfer [62–64] (Figure 2B). Parkin autoinhibition is also enforced by a short repressor element (REP) located between the IBR and RING2 domains; mutations in the REP region relieve autoinhibition by allowing E2 access [57,61,64]. Additionally, the catalytic cysteine within RING2 is buried through interactions with RING0, further contributing to autoinhibition [57,61–64]. Structural disruption of this RING0–RING2 interface or deletion of RING0 results in activation of Parkin [57,61,62]. Another layer of autoinhibition involves the burial of the donor ubiquitin-binding site located across the IBR and RING1 domains, as revealed in later studies [65].

## Activating Parkin: the allosteric route

A major breakthrough in understanding Parkin activation came with the discovery that phosphorylation of ubiquitin at Ser65 by PINK1 acts as a potent allosteric activator of Parkin [42,47,48,50,66]. In response to mitochondrial damage, PINK1 accumulates on the outer mitochondrial membrane and phosphorylates ubiquitin, initiating Parkin recruitment and activation [67–73]. Nearly a decade later, a similar phosphorylation event was observed on NEDD8, a ubiquitin-like protein, which also led to Parkin activation [74]. However, in contrast with pUb, the physiological role of phospho-NEDD8 (pNEDD8) remains unclear.

pUb recruits cytosolic Parkin to the mitochondria, where Parkin itself is phosphorylated at its Ubl domain on Ser65, further enhancing its activation [43,50,53]. Ordureau and colleagues proposed a positive feedback mechanism in which pUb binding promotes Parkin recruitment to damaged mitochondria, where Parkin is phosphorylated and catalyzes ubiquitination of mitochondrial substrates [54]. These ubiquitin chains are subsequently phosphorylated by PINK1, generating more pUb and thereby amplifying the mitochondrial signaling cascade [54,75].

Following the discovery of pUb, several studies aimed to uncover the molecular mechanism behind pUb-mediated Parkin activation. Earlier biophysical experiments showed that the Ubl domain could bind in trans to ΔUbl Parkin (lacking the Ubl domain), reinforcing Parkin’s autoinhibited conformation [59]. However, pUb binding disrupted this interaction, indicating that pUb displaces the Ubl domain, relieving autoinhibition and promoting activation [63,64,76]. These findings led to a model in which pUb binding triggers conformational rearrangement by displacing the Ubl domain, thereby activating Parkin.

**Figure 3:**
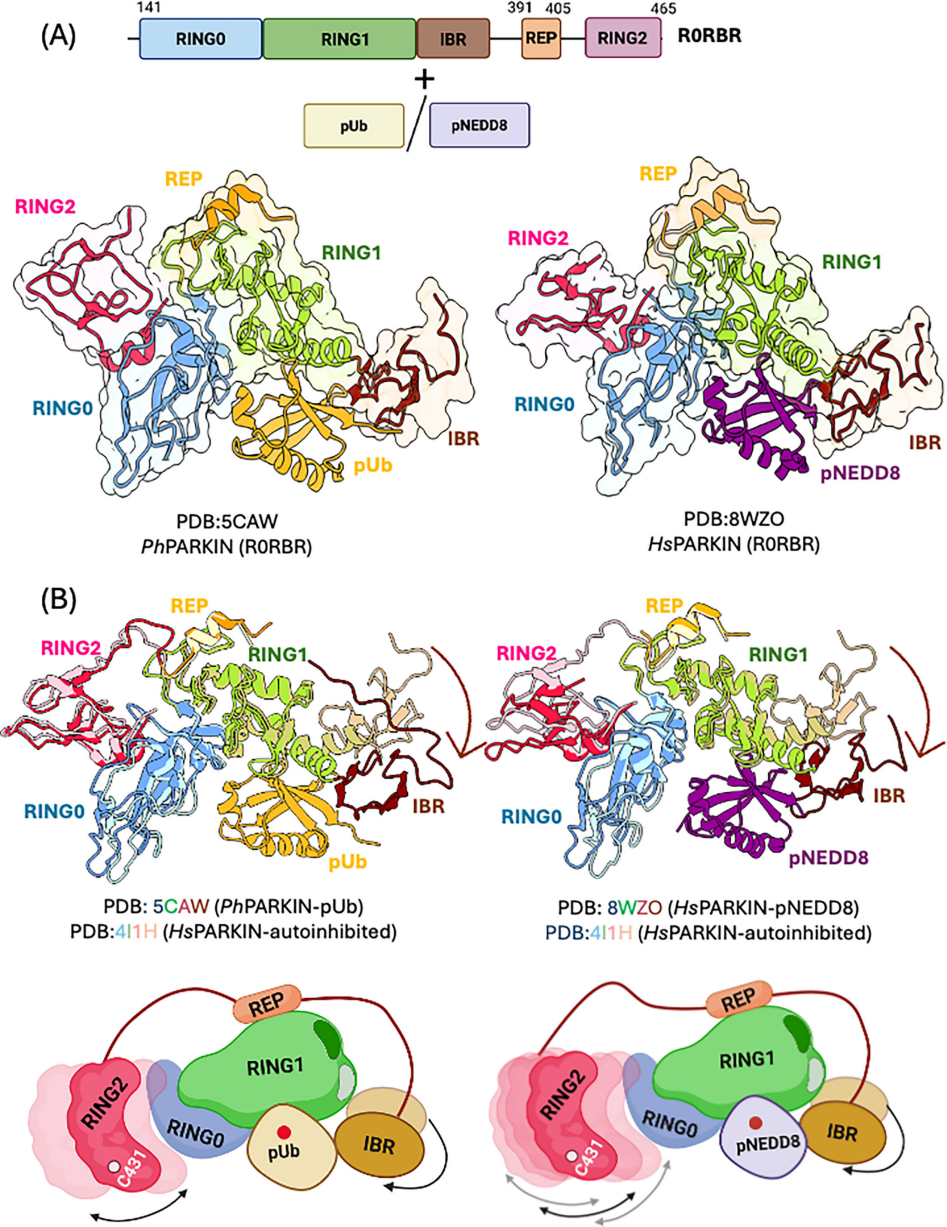
Conformational changes in Parkin upon binding to pUb or pNEDD8. **(A**) Schematic of the Parkin R0RBR construct used for crystallizing the Parkin–pUb/pNEDD8 complexes (upper panel). Crystal structure of pUb and pNEDD8 bound Parkin (PDB:5CAW, PDB:8WZO) (bottom panels left and right).(**B**) Structural comparison of apo Parkin R0RBR (PDB:4I1H) with pUb-bound (PDB: 5CAW) and pNEDD8-bound (PDB: 8WZO) forms. Schematic summarizing the conformational changes in Parkin observed upon pUb or pNEDD8 binding (lower panel).

Further insights into the mechanism of pUb-mediated Parkin activation came from structural studies. The first crystal structure of Parkin’s R0RBR fragment bound to pUb revealed a basic patch (K151, H302, R305) that forms the pUb-binding interface (Figure 3A) [76]. However, this structure did not show expected conformational changes, such as displacement of the repressor element (REP) or RING2 domain. Similarly, Kumar and colleagues solved the structure of human Parkin (UblR0RBR) bound to pUb, which showed that the Ubl domain remained associated and no significant structural rearrangements of REP/RING2 were observed compared with the apo state [65]. However, the study identified a donor ubiquitin (E2–Ub_donor_)-binding site at the IBR–RING1 interface that was buried in the inactive Parkin conformation, suggesting partial activation [65]. Interestingly, comparison with the structure of Parkin bound to pNEDD8 revealed that RING2 adopted a more dynamic state compared with its pUb-bound conformation [77], indicating a potential difference in the degree of activation conferred by these phosphorylated ligands (Figure 3A–B). Moreover, biochemical and biophysical analyses showed that pNEDD8 binds Parkin with significantly higher affinity than pUb, which was attributed to favorable sequence and structural features at the interaction interface [77]. For example, the N(Ub)60K(NEDD8) and Q(Ub)62L(NEDD8) substitutions in NEDD8 enable hydrogen bonding between NEDD8 K60 and the carbonyl group of Parkin L187, as well as hydrophobic interactions between NEDD8 L62 and Parkin V224 [77].

A novel pUb-binding site, comprising K161, R163, and K211, was later proposed on the RING0 domain, in addition to the previously known interface [78,79]. This suggested a degree of promiscuity in how pUbl proteins interact with Parkin. Functional data supported the relevance of this new site: the K211N mutation in RING0 impaired pUb-mediated Parkin activation and mitochondrial recruitment [76,79]. A revised feedforward model was thus proposed, where pUb binding to RING0 is preferred over pUbl, contributing more significantly to Parkin activation [78,79].

A new observation emerged from studies involving pNEDD8: Lenka and colleagues found that the K211N mutation also abolished pNEDD8-mediated activation, despite its higher binding affinity [77]. Structural analysis revealed that K211N induced a conformational change in RING0 that impaired Parkin function independently of pUb or pNEDD8 binding, highlighting a critical role for RING0 conformation in allosteric activation [77].

Overall, while pUb and pNEDD8 binding induce relatively modest conformational shifts in Parkin, these are sufficient for partial activation. However, full activation probably requires synergistic phosphorylation of Parkin’s own Ubl domain. These findings continue to refine our understanding of Parkin’s activation mechanism and point to multiple layers of regulation involving dynamic domain rearrangements.

## pUbl-mediated Parkin activation: deciphering the cis vs. trans puzzle

Phosphorylated Parkin (phospho-Parkin) exhibited significantly higher enzymatic activity compared with its activation by allosteric modulators such as pUb or pNEDD8 [45–48,50,63–65,74,76,77,80]. This observation was consistent with structural data showing that, in Parkin–pUb and Parkin–pNEDD8 complexes, the repressor element (REP) and RING2 domains remained in a closed conformation, associated with the Parkin core [65,76,77,80]. These structures suggested that allosteric activators alone do not induce full activation.

In the absence of crystal structures of phospho-Parkin at the time, early mechanistic models were driven by computational and biophysical studies. A computational study by Caulfield et al. [81] proposed that phosphorylation of the Ubl domain leads to its displacement, relieving autoinhibition. Biochemical studies showed that while the unmodified Ubl domain interacts with ΔUbl Parkin in trans, the pUbl domain exhibits weak or no binding to the Parkin core [63,64,76]. These findings supported a model in which phosphorylation of the Ubl domain causes its release from RING1, thereby activating Parkin.

**Figure 4:**
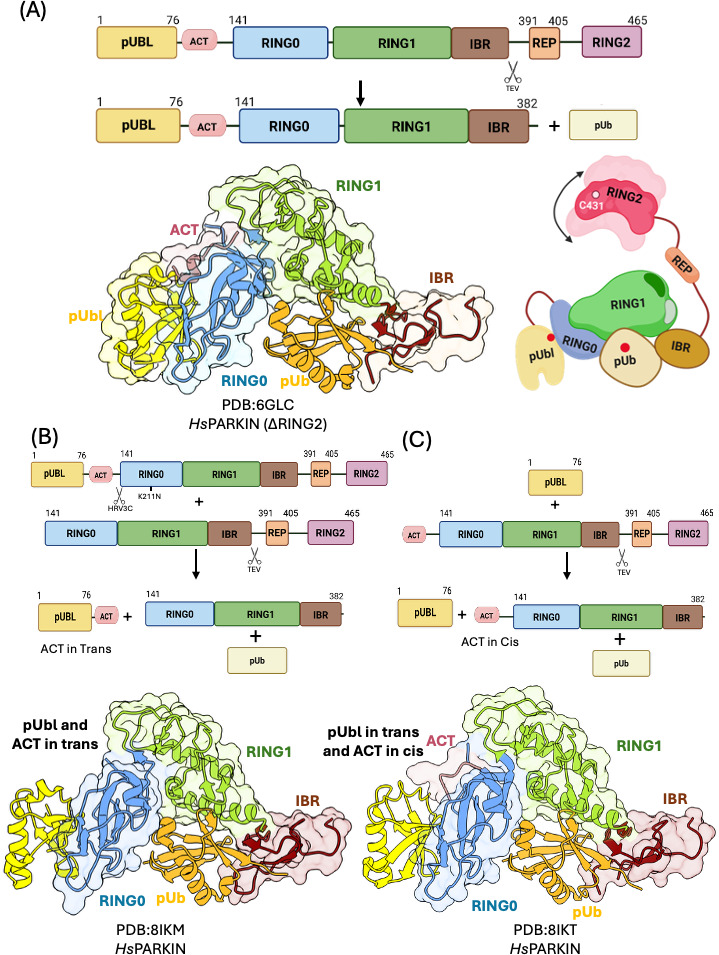
Crystal structures of phospho-Parkin–pUb complexes in cis and trans states. **(A)** A schematic representation of the domain boundaries of the phospho-Parkin construct and strategy used for crystallization of phospho-Parkin in cis (upper panel). Crystal structure of phospho-Parkin–pUb complex (PDB: 6GLC) (left panel). A schematic representation illustrating conformational changes upon Parkin phosphorylation in cis (right panel). **(B)** A schematic representation of the domain boundaries of the Parkin constructs and strategy used for crystallization of phospho-Parkin in trans (pUbl and ACT expressed separately) (upper panel). Crystal structure of phospho-Parkin–pUb complex in trans (pUbl and ACT expressed separately) (PDB: 8IKM, lower panel). **(C)** A schematic representation of the domain boundaries of the Parkin constructs and strategy used for crystallization of phospho-Parkin in trans (ACT in cis) (upper panel). Crystal structure of phospho-Parkin–pUb complex in trans (ACT in cis) (PDB:8IKT lower panel).

Structural breakthroughs came in 2018 with the determination of phospho-Parkin structures lacking REP and RING2 domains (Figure 4A) [45,46]. These studies revealed that, upon phosphorylation, the Ubl domain does not merely dissociate from RING1, but instead binds to a basic patch on the RING0 domain composed of K161, R163, and K211 [45,46]. Importantly, these publications also showed displacement of the REP–RING2 module from the Parkin core, an essential step toward full activation. Additionally, Gladkova et al. [45] identified a previously uncharacterized activating element (ACT) within the linker region between the Ubl and RING0 domains (Figure 4A). This element was found to interact with RING0 in the activated conformation, and its deletion or mutation impaired Parkin activity, highlighting its functional importance [45].

Both phospho-Parkin structures published in 2018 captured the pUbl domain interacting with RING0 in cis, within the same Parkin molecule [45,46]. This finding was in apparent contrast to earlier trans-binding studies, in which pUbl failed to interact with ΔUbl Parkin [63,64,76]. A key experimental difference was that the earlier trans-binding assays were performed with intact REP–RING2 domains, while the cis structures were obtained using either truncated constructs lacking REP–RING2 or through proteolytic removal of these domains after phosphorylation. To directly address this discrepancy, Lenka and colleagues employed a similar strategy [45] to monitor the displacement of REP–RING2 domains and pUbl interaction in trans [80]. They demonstrated that pUbl could bind to the RING0 domain of Parkin and displace REP–RING2 in trans as well. Moreover, they showed that the ACT element could also complement activation in trans, although it was more effective in cis, consistent with structural observations showing ordered ACT in cis and disordered ACT in trans (Figure 4B–C) [80].

Interestingly, while biophysical data confirmed that phospho-Parkin can interact with other Parkin molecules both in cis and trans, catalytic activity assays showed that fully phosphorylated Parkin is significantly more active than mixtures where catalytically dead phospho-Parkin was titrated into wildtype Parkin [80]. These findings suggest that while phospho-Parkin can promote trans activation of Parkin, its catalytic efficiency in this context is markedly low. However, the data do not exclude several contributing complexities. First, catalytically inactive phospho-Parkin may still bind E2–Ub, potentially acting as a competitive inhibitor of native Parkin. Second, the higher effective local concentration of RING2 within the native Parkin molecule could outcompete transacting pUbl, thereby limiting overall activity in the assay setup. Thus, although cis activation appears as the primary mechanism of Parkin activation, a weaker trans activation driven by pUbl-mediated interactions between Parkin molecules may be important for activating Parkin isoforms that lack either the Ubl domain or the RING2 domain [80]. Additionally, individuals carrying heterozygous Parkin mutations, one normal allele and one mutant, may still compensate for functional deficits through trans activation of Parkin [80].

## Concluding remarks

**Figure 5:**
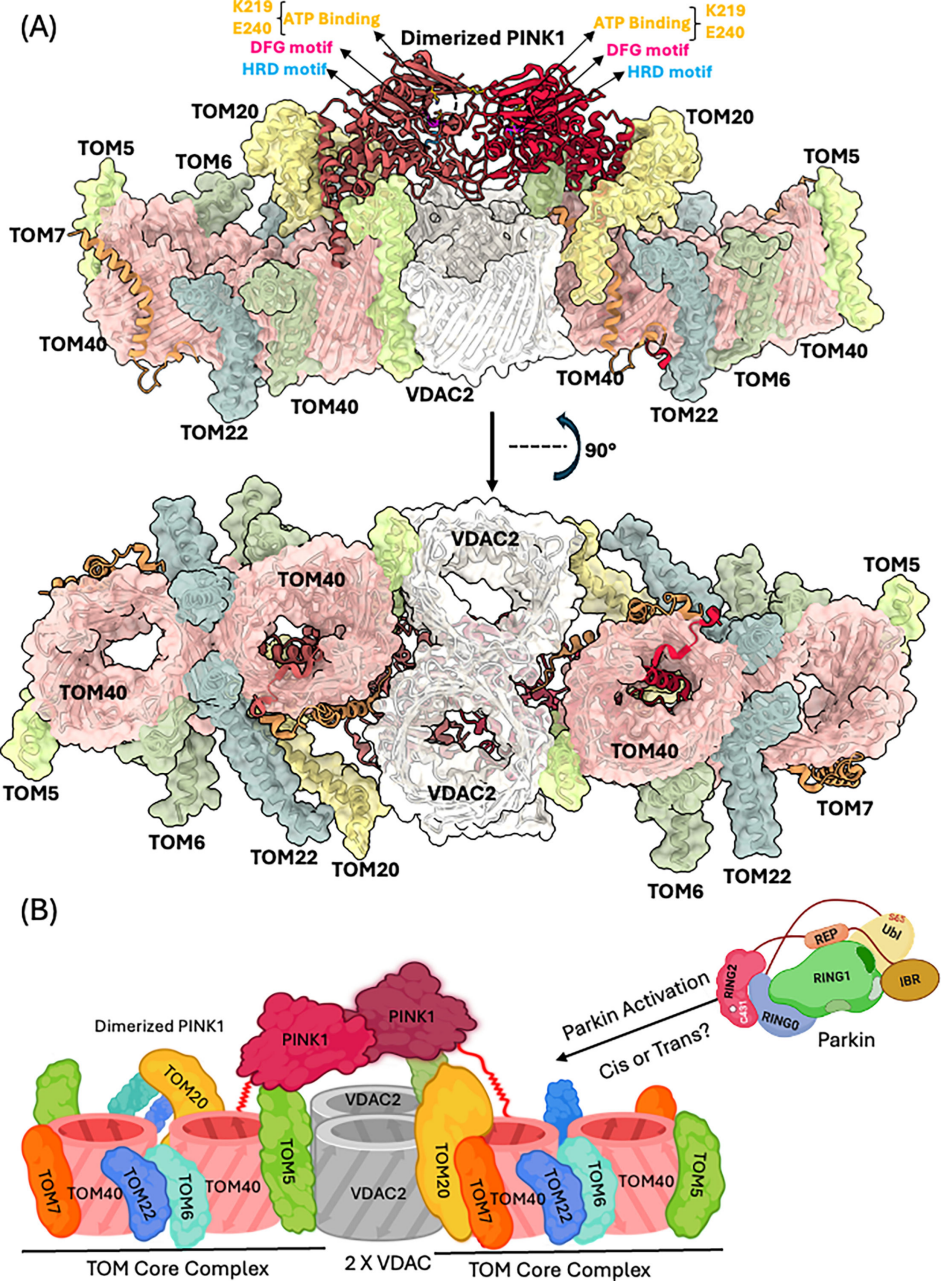
Structural insights into PINK1–mitochondria association, the initial step in Parkin activation. **(A**) Cryo-EM structure of PINK1 bound to the mitochondrial membrane (PDB: 9EIH). (**B**) Schematic representation of panel A, outlining key features and identifying directions for future research in the Parkin activation pathway.

Over the past decade, significant progress has been made in understanding the PINK1–Parkin-mediated mitophagy pathway, particularly the mechanisms underlying activation of Parkin’s E3 ubiquitin ligase activity. With the emergence of new structural and biochemical data, multiple models of Parkin activation have been proposed and refined. A major recent advancement is the structural elucidation of human PINK1 at the mitochondrial TOM–VDAC complex, which provides critical insights into the earliest steps of Parkin activation, beginning with PINK1 recruitment to the mitochondrial surface [82]. Notably, the structure captures PINK1 as a dimer bound to the TOM–VDAC array (Figure 5), suggesting a regulated assembly at the mitochondrial entry point. Notably, PINK1’s kinase activity depends on trans-autophosphorylation [83], a process that parallels Parkin activation in trans. However, key questions remain unanswered. It remains to be determined how Parkin is recruited downstream of PINK1 and whether this process is mediated through cis or trans interactions similar to those seen with PINK1. Additionally, the functional significance of Parkin isoforms lacking the Ubl or RING2 domains [80] remains unclear, specifically whether these truncated forms can assemble into functional Parkin complexes and contribute to mitophagy signaling. Moreover, the mechanism by which Parkin recognizes its substrates remains to be elucidated.

**Summary**

- Mutations in the genes encoding PINK1 kinase and Parkin E3 ubiquitin ligase lead to autosomal recessive juvenile Parkinsonism (AR-JP).
- Parkin is an autoinhibited enzyme, and its activation requires phosphorylation of both ubiquitin/NEDD8 and Parkin itself by PINK1.
- Binding of phosphorylated ubiquitin or NEDD8 (pUb/pNEDD8) in the cleft between the RING0 and RING1 domains induces partial activation of Parkin through allosteric conformational changes.
- Full activation of Parkin occurs when the phosphorylated Ubl (ubiquitin-like) domain binds to RING0, near the catalytic RING2 domain.
- Biophysical studies have shown that the pUbl domain can interact with Parkin in both cis (same molecule) and trans (separate molecules) configurations. However, confirmation of these mechanisms on mitochondria and their physiological relevance is still pending.

## Open Access

This article has been published open access under our Subscribe to Open programme, made possible through the support of our subscribing institutions, learn more here: https://portlandpress.com/pages/open_access_options_and_prices#conditional

## Abbreviations

AR-JP: autosomal recessive juvenile Parkinsonism
PD: Parkinson’s disease
PINK1: PTEN-induced kinase 1
PTEN: phosphatase and tensin homolog
Ubl: ubiquitin-like
